# Outcomes of percutaneous transhepatic gallbladder drainage versus percutaneous transhepatic biliary drainage for obstructive jaundice

**DOI:** 10.1371/journal.pone.0310469

**Published:** 2025-02-24

**Authors:** Tetsushi Azami, Yuichi Takano, Naoki Tamai, Jun Noda, Masataka Yamawaki, Fumitaka Niiya, Naotaka Maruoka, Fumiya Nishimoto, Akira Ishihara, Masatsugu Nagahama

**Affiliations:** 1 Department of Medicine, Division of Gastroenterology, Showa University Fujigaoka Hospital, Yokohama, Japan; 2 Department of Gastroenterological Surgery, Hitachi Medical Center, Hitachi, Japan; Universitatsklinikum Leipzig, GERMANY

## Abstract

Percutaneous transhepatic gallbladder drainage (PTGBD) is an alternative to percutaneous transhepatic biliary drainage (PTBD) for cases with obstructive jaundice in which the bile duct obstruction is below the confluence of the cystic ducts. This retrospective study aimed to evaluate the usefulness of PTGBD and PTBD in patients with obstructive jaundice. We recruited patients who had undergone percutaneous biliary drainage for acute cholangitis and obstructive jaundice at two institutions between January 2017 and March 2024. In principle, PTBD was the first choice. PTGBD was selected for cases where the intrahepatic bile duct diameter was ≤ 5 mm or ≥ 6 mm with significant respiratory-related variability of the positioning of the bile ducts. In other cases, PTBD was chosen. Fifty-five patients were included in this analysis. However, patients with intrahepatic or hilar bile duct stenosis, post choledocholithiasis, complex cholecystitis, total bilirubin levels of < 2.0 mg/dL, and uncorrectable bleeding tendency and those who had undergone the procedure and later discontinued without puncture were excluded. The technical success rates, clinical success rates, and complication rates of the procedure were evaluated. The technical success rates were 96.3% (26/27) and 82.1% (23/28) in the PTGBD and PTBD groups, respectively. The clinical success rates were 85.2% (23/27) and 67.9% (19/28) in the PTGBD and PTBD groups, respectively. The complication rates were 18.5% (5/27) and 25.0% (7/28) in the PTGBD and PTBD groups, respectively. No serious complications were observed in either group. Hence, the two groups did not significantly differ in any of the endpoints. The outcomes of PTGBD were comparable to those of PTBD in patients with obstructive jaundice. Hence, PTGBD is a reasonable treatment option for cases of obstructive jaundice in which PTBD is not feasible.

## Introduction

Bile duct drainage is indicated for all patients with acute cholangitis and obstructive jaundice, except for some mild cases that improve with antimicrobial therapy alone. The Tokyo Guidelines 2018 recommend endoscopic biliary drainage (EBD) as the first-line treatment for bile duct drainage [1]. However, in some cases, EBD cannot be performed due to factors such as the patient’s general condition, postoperative reconstructed intestinal tract, and gastrointestinal stenosis. In recent years, endoscopic ultrasound-guided biliary drainage (EUS-BD) has been the salvage procedure of choice for such cases. If EBD and EUS-BD are challenging to perform, percutaneous transhepatic biliary drainage (PTBD) is considered. However, its technical success rate is 63% in patients with nondilated bile ducts and 86% in patients with dilated ducts, which is not sufficient [2].

Alternatively, percutaneous transhepatic gallbladder drainage (PTGBD) is a drainage technique for acute cholecystitis. It has a high success rate and can be useful for bile duct drainage if the bile duct obstruction is downstream of the cystic duct. EUS-guided gallbladder drainage has been reported to be useful for EUS-guided hepaticogastrostomy failure in obstructive jaundice [3]. PTGBD can serve as an alternative treatment strategy to PTBD. A case series and a small number of retrospective cohort studies have evaluated the use of PTGBD for cholangitis and obstructive jaundice [4–7]. However, to the best of our knowledge, no studies have compared PTGBD and PTBD. The present study aimed to examine the usefulness of PTGBD and PTBD in patients with obstructive jaundice.

## Materials and methods

### Study design and participants

This retrospective study was based on an analysis of data collected from the patients’ electronic medical records. Patients who had undergone percutaneous biliary drainage for acute cholangitis and obstructive jaundice at Showa University Fujigaoka Hospital or Hitachi Medical Center between January 1, 2017 and March 31, 2024 were included in this analysis. Patients with intrahepatic or hilar bile duct stenosis, hepaticojejunostomy anastomotic stricture, coexistence of cholecystitis, total bilirubin (T.bil) level of < 2.0 mg/dL, and uncorrectable bleeding tendency and those who had undergone the procedure and later discontinued without puncture were excluded.

Treatment after PTGBD/PTBD was decided based on the improvement in cholangitis and overall condition. Surgery and EUS-BD were considered in cases of difficult biliary cannulation. Treatment via percutaneous drainage route was also considered, in which case, a waiting period of 1–2 weeks was enforced to ensure adequate fistula formation. When the patient’s general condition was poor, the risk of the next procedure was considered high, or when the prognosis was considered short, the catheter was left in place permanently.

The data were accessed for research purposes on August 16, 2024. The study and all of its protocols conform with and were conducted according to the guidelines set in the Declaration of Helsinki and were approved by the Showa University Research Ethics Review Board (Approval Number: 2024-106-B). The need for informed consent was waived by the IRB due to the retrospective nature of the study.

### Outcomes and definitions

The primary endpoints were technical success rates, clinical success rates, and complication rates. Technical success was defined as catheter placement into the biliary tract. Clinical success was defined as a decrease in T.bil levels to < 2.0 mg/dL or a 50% decrease in T.bil levels after 2 weeks. Complications were defined as “early complications” when they occurred within 14 days of surgery, and “late complications” when they occurred 15 or more days postoperatively. Complications were evaluated using the Clavien–Dindo classification [8]. The severity of cholangitis was also assessed using the Tokyo Guidelines 2018 [1]. Time to PTGBD was defined as the duration (in hours) from initial consultation to the day of the procedure.

### Procedure details

**Fig 1:**
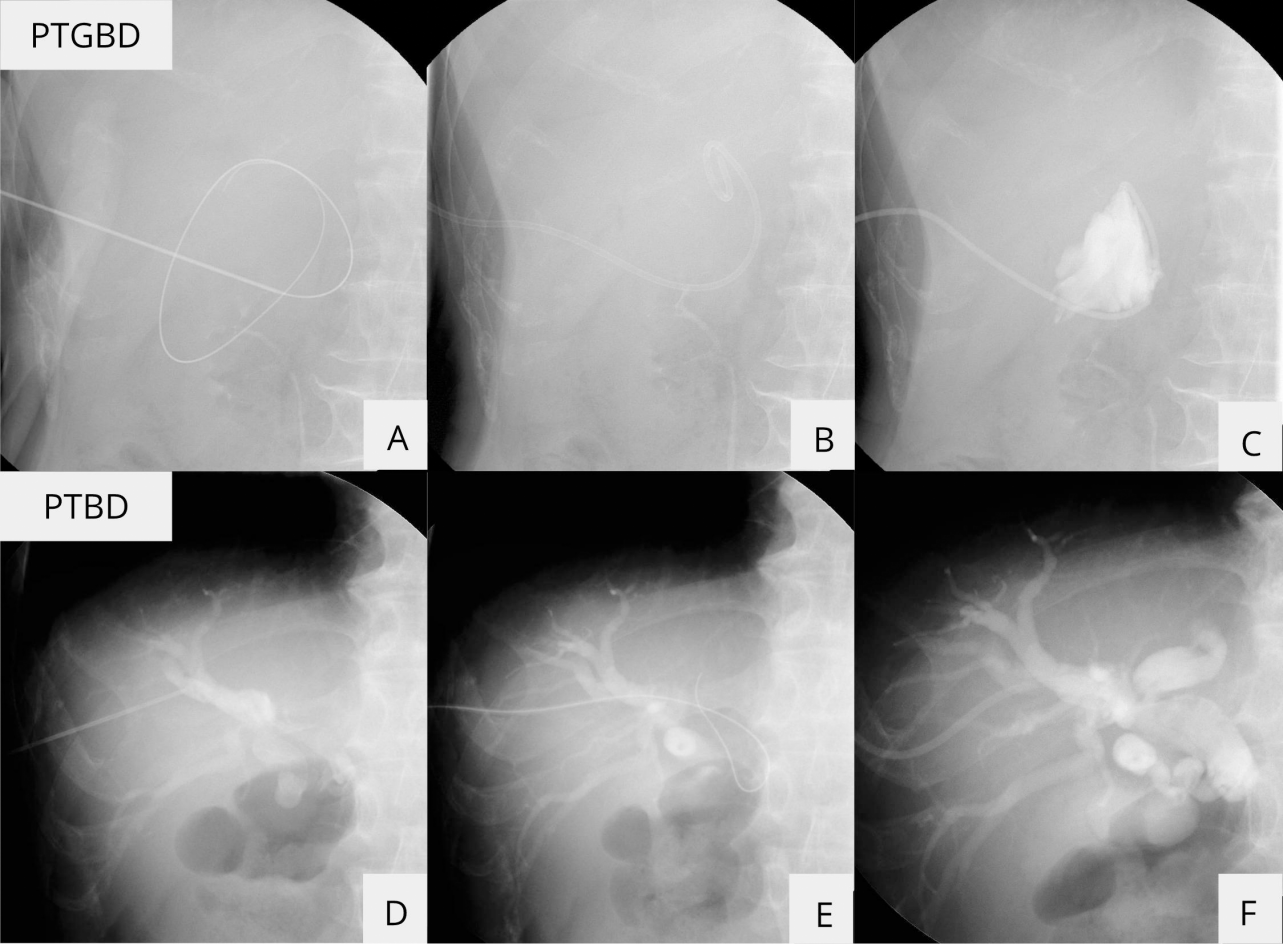
Schematic of the percutaneous transhepatic gallbladder drainage and percutaneous transhepatic biliary drainage. A) An 18-G needle was inserted into the gallbladder. Next, a 0.035-in guide wire was placed in the gallbladder. (B) An 8-Fr pigtail catheter was inserted. (C) Cholangiography was performed. (D) An 18-G needle was inserted into the gallbladder and cholangiography was performed. (E) A 0.035-in guide wire was placed in the bile duct. (F) An 8-Fr pigtail catheter was inserted.

In principle, PTBD was the first choice of therapy, but PTGBD was selected for cases where the intrahepatic bile duct diameter was ≤ 5 mm or ≥ 6 mm with significant respiratory-related variability of the positioning of the bile ducts. A one-step technique was used for PTGBD, and PTBD was performed using a one- or two-step technique, at the discretion of the attending surgeon (Fig 1). The tip of the catheter was placed in the bile duct for external drainage in both PTGBD and PTBD. If necessary, analgesics (pentazocine 15 mg) were administered intravenously before puncture.

#### One-step method.

After administering local anesthesia with lidocaine, an 18-G needle (Hanaco Medical, Saitama, Japan) was inserted into the gallbladder or bile duct under ultrasound guidance, and a small amount of bile was aspirated. Next, a 0.035-in guide wire (Radifocus, Terumo, Tokyo, Japan) was placed in the gallbladder or bile duct under fluoroscopy. After a small skin incision was made and dilated with a dilator, a 6–8-Fr pigtail catheter (UreSil, L.C.C., Illinois, USA) was inserted.

#### Two-step method.

After administering local anesthesia with lidocaine, a 20-G needle was inserted under ultrasound guidance. A small amount of bile was aspirated. Then, cholangiography was conducted (Urografine 60%, Byer AG, Nordrhein-Westfalen, Germany). Next, a 0.018-in guide wire was placed in the bile duct under fluoroscopic guidance. A small skin incision was made, an introducer set was inserted, and all but the sheath was removed. Then, a 0.035-in guide wire was inserted, the sheath was removed, and the bile duct was dilated with a dilator. Finally, a 7-Fr pigtail catheter (CLINY PTCD Kit, Create Medic Co., Yokohama, Japan) was implanted.

### Statistical analysis

The outcomes of the PTGBD and PTBD groups were compared using the Mann–Whitney U test for continuous variables and chi-square test or Fisher’s exact test for nominal variables. A p value of < 0.05 was considered statistically significant. R version 4.0.3 (R Foundation for Statistical Computing, Vienna, Austria) was used for data analysis.

## Results

### Clinical characteristics of the patients

Percutaneous drainage was performed on 79 patients with cholangitis and obstructive jaundice between January 1, 2017, and March 31, 2024. Among them, two presented with hilar bile duct stenosis, seven with hepaticojejunostomy anastomotic stricture, seven with coexisting cholecystitis, and eight with T.bil levels <  2.0 mg/dL were excluded. Hence, 55 patients were finally included in this study. The number of cases who experienced failure of cannulation during ERCP was 4 in the PTGBD group and 13 in the PTBD group. Percutaneous drainage for surgically altered anatomy, duodenal invasion, respiratory failure or septic shock was performed without ERCP in 13, 3, and 7 patients from the PTGBD group, respectively; and 10, 5, and 0 patients from the PTBD group, respectively.

**Fig 2:**
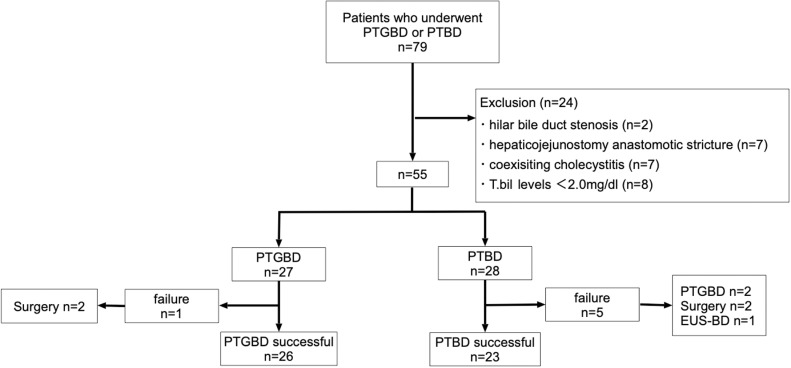
Flow chart of participant enrollment. PTGBD: percutaneous transhepatic gallbladder drainage. PTBD: percutaneous transhepatic biliary drainage. EUS-BD: endoscopic ultrasound-guided biliary drainage.

A total of 27 patients (25 with intrahepatic bile duct diameters ≤ 5 mm or less and 2 with significant respiratory-related variability of positioning of the bile ducts) underwent PTGBD, and 28 patients with an intrahepatic bile duct diameter of ≥ 6 mm and small respiratory variability underwent PTBD. One patient in the PTGBD group and five in the PTBD group experienced technical failure. Technical success was achieved in 26 and 23 participants from the PTGBD and PTBD groups, respectively (for PTBD, this related to 21 who underwent the one-step procedure and 2 who underwent the two-step procedure) (Fig 2).

In terms of technical failures, in one patient, PTGBD was unsuccessful because the dilator could not be inserted due to the lack of gallbladder distension. Surgical treatment was then performed. In five patients in whom PTBD failed, three and two underwent PTGBD and EUS-BD, respectively (Fig 2). Table 1 shows the characteristics of the patients. The PTGBD and PTBD groups did not significantly differ in terms of age, sex, use of antithrombotic medication, and duration of antibiotic therapy. The median Charlson Comorbidity Index (CCI) values were 7 (3–12) in the PTGBD group and 9 (4–11) in the PTBD group. The PTBD group (9.33 [2.0–26.2] mg/dL) had significantly higher baseline T.bil levels than the PTGBD group (4.30 [2.3–22.7] mg/dL). The mean diameter of the intrahepatic duct was significantly larger in the PTBD group (20 [7–16] mm) compared with the PTGBD group (3 [1–11] mm). Severe cholangitis was significantly more common in the PTGBD group than in the PTBD group (14 [51.9%] vs 5 [17.9%]). The mean time to procedure was significantly shorter in the PTGBD group (7 [3–144] hours) compared with the PTBD group (75 [6–336] hours). In terms of obstruction etiology, 18 (66.7%) and 9 (33.3%) patients in the PTGBD group presented with benign and malignant diseases, respectively. Moreover, 5 (17.8%) and 23 (82.1%) patients in the PTBD group presented with benign and malignant diseases, respectively. Hence, benign disease was more common in the PTGBD group than in the PTBD group. The most common reasons for failure or absence of endoscopic retrograde cholangiopancreatography (ERCP) were postoperative surgically altered anatomy in the PTGBD group (n =  13 [48.1%]) and failed cannulation in the PTBD group (n =  13 [46.4%]). The length of hospital stay was significantly longer in the PTBD group (55.5 [15–129] days) compared with the PTBD group (23 [8–65] days) (S1 Table).

**Table 1:** Comparison of participant characteristics in relation to drainage type.

Characteristics	PTGBD (n = 27)	PTBD (n = 28)	*P*-value
Age (years), median (range)	76 (53–93)	73 (59–95)	0.34
Sex (male), n (%)	20 (74.1)	18 (64.3)	0.684
Charlson Comobidity Index, median (range)	7 (3–12)	9 (4–11)	0.0599
Use of antithrombotic medication, n (%)	5 (18.5)	4 (14.2)	1
Baseline T.bil levels (mg/dl), median (range)	4.30 (2.3–22.7)	9.33 (2.0–26.2)	< 0.01
Diameter of intrahepatic duct (mm), median (range)	3 (1–11)	10 (7–16)	< 0.01
Severe cholangitis, n (%)	14 (51.9)	5 (17.9)	0.0111
etiology of obstruction			
Benign, n (%)	18 (66.7)	5 (17.8)	< 0.01
Malignant, n (%)	9 (33.3)	23 (82.1)	
Bile duct stone	17	5	
Chronic pancreatitis	1	0	
Pancreatic cancer	4	12	
Bile duct cancer	1	6	
Ampullary cancer	1	1	
Gastric cancer	3	3	
Malignant lymphoma	0	1	
Reason for ERCP failed or not done, n (%)			< 0.01
Surgically altered anatomy	13 (48.1)	10 (35.7)	
Duodenal invasion	3 (11.1)	5 (17.9)	
Failed cannulation	4 (14.8)	13 (46.4)	
Respiratory failure or septic shock	7 (25.9)	0	
Time to procedure (hour), median (range)	7 (3–144)	75 (6–336)	< 0.01
Duration of antibiotics therapy (day), median (range)	14 (5–28)	12 (2–37)	0.428
Length of hospital stay (day), median (range)	23 (8–65)	55.5 (15–129)	< 0.01

PTGBD: percutaneous transhepatic gallbladder drainage. PTBD: percutaneous transhepatic biliary drainage. ERCP: endoscopic retrograde cholangiopancreatography. T.bil: total bilirubin.

### Outcomes

The technical success rates were 96.3% (26/27) in the PTGBD group and 82.1% (23/28) in the PTBD group. The clinical success rates were 85.2% (23/27) in the PTGBD group and 67.9% (19/28) in the PTBD group. Hence, the two groups did not significantly differ in terms of technical success and clinical success rates. Although not included in the PTBD group, one patient who did not achieve clinical success with PTGBD achieved clinical success for PTBD. Rates of early complications were 11.1% (3/27) and 17.9% (5/28) in the PTGBD and PTBD groups, respectively. Rates of late complication were 7.41% (2/27) and 7.14% (2/28) in the PTGBD and PTBD groups, respectively. There were no significant differences in terms of the rates of complication between the two groups (Table 2).

**Table 2:** Comparison of outcomes in relation to drainage type.

	PTGBD (n = 27)	PTBD (n = 28)	*P*-value
Technical success rates, % (n)	96.3 (26)	82.1 (23)	0.193
Clinical success rates, % (n)	85.2 (23)	67.9 (19)	0.205
Complication rates, %	18.5	25	0.748
Early complications rates, % (detail)	11.1 (catheter miglation: n = 2, shock grade Ⅱ: n = 1)	17.9 (catheter miglation: n = 3, shock grade Ⅱ: n = 1, bleeding grade Ⅰ: n = 1)	0.705
Late complications rates, % (detail)	7.41 (catheter miglation: n = 1, catheter insertion site infection gradeⅡ: n = 1)	7.14 (catheter obstruction grade Ⅰ: n = 2)	1

PTGBD: percutaneous transhepatic gallbladder drainage. PTBD: percutaneous transhepatic biliary drainage

Additional treatments performed after PTBD or PTGBD were ERCP (13), other surgery (2), PTBD (1), and permanent catheter placement (11) in the PTGBD group; percutaneous treatment (12), rendezvous procedure (8), percutaneous bile duct stenting (3), percutaneous bile duct stone removal (1), PTGBD (2), other surgery (2), EUS-BD (1), and permanent catheter placement (14) in the PTBD group. The median durations of catheter placement (among in patients for whom it could be removed) were 21 and 41.5 days in the PTGBD and PTBD groups, respectively, with no significant difference (p value =  0.19).

## Discussion

Cholecystocentesis, which was first reported by Burckhardt and Muller in 1921, has a long history [9]. Subsequently, Huard and Du-Xuan-Hop et al. successfully performed intrahepatic bile duct puncture in 1937 [10]. The introduction of echo-guided puncture and improvements in treatment tools led to therapeutic application of percutaneous biliary puncture. PTBD was first reported by Glenn et al. in 1962 [11]. PTGBD was first reported by Elyaderani and Gabriele in 1979 [12] and performed by Radder in 1982 in patients with acute cholecystitis [13]. To date, in the Tokyo Guidelines, PTGBD is recommended as the standard drainage method for patients with cholecystitis who are at high risk for surgery, and PTBD is an alternative treatment to EBD in patients with cholangitis [1].

In recent years, EUS-BD has been gaining popularity as a biliary drainage technique since the study of Giovannini [14]. Moreover, EUS-GBD has been reported to be useful in treating obstructive jaundice in cases of EUS-guided hepaticogastrostomy failure if the bile duct obstruction was downstream of the confluence of the cystic duct. Furthermore, PTGBD may be useful in obstructive jaundice. A case series and a few retrospective cohort studies have long documented the efficacy of PTGBD for obstructive jaundice [4–7].

Li et al. and Park et al. reported that PTGBD resulted in jaundice control in 91% (29/32) and 100% (20/20) of patients with obstructive jaundice. Only 3% (one patient with biliary peritonitis) and 5% (one patient with catheter migration) of patients presented with complications, respectively. Technical success was 100% in both studies. Both studies have revealed that PTGBD is useful in treating obstructive jaundice [4,5].

No reports have compared PTGBD and PTBD. This is the first report comparing the efficacy of PTGBD and PTBD in obstructive jaundice. The technical success, clinical success, and complication rates of PTGBD and PTBD were comparable in this study. PTGBD for obstructive jaundice should be considered as an alternative treatment to PTBD. However, in one case of PTBD, jaundice improved when no clinical response to PTGBD was achieved. Treatment via the percutaneous drainage route (rendezvous procedure, percutaneous bile duct stenting, and stone removal) is also less difficult with PTBD than with PTGBD. In fact, the present study found 12/28 patients who underwent PTBD group underwent treatment via the percutaneous drainage route. On the other hand, no patients in the PTGBD group underwent treatment via percutaneous drainage. For patients considering percutaneous drainage, PTBD could represent a better option because of the ease of subsequent procedures. However, treatment via percutaneous drainage often takes longer, requires multiple sessions, and involves longer hospital stays.

Although there were no significant differences in terms of outcomes between the two groups, the clinical characteristics of the two groups differed. In terms of disease etiology, benign diseases such as common bile duct stones were more common in the PTGBD group than in the PTBD group. Meanwhile, malignant diseases were more common in the PTBD group than in the PTGBD group. Severe cholangitis was significantly more common in the PTGBD group than in the PTBD group. Severe cholangitis caused by bile duct stones requires a short treatment time due to unstable vital signs. In several cases, it is difficult to obtain patient cooperation and respiratory arrest at the time of puncture. Therefore, PTGBD, which is less difficult to perform, was selected. Thus, PTGBD was used for emergency drainage in many cases with severe cholangitis, and the time to procedure was significantly shorter in PTGBD. When PTGBD was selected under such circumstances, ERCP was often performed after improvement of cholangitis (13/27 cases). Therefore, the number of cases that eventually required permanent catheter placement was lower among participants who underwent PTGBD compared with PTBD (40.7% (11/27) in the PTGBD group vs. 50% (14/28) in the PTBD group). By contrast, obstructive jaundice caused by malignant diseases is generally more severe than that caused by benign disease. However, it is less likely to be complicated by severe cholangitis. The high degree of bile duct dilatation and the ease of patient cooperation might have been the reasons for selecting PTBD. Thus, there were many cases of mild/moderate cholangitis in the PTBD group, and ERCP was attempted before PTBD was performed. However, biliary cannulation was unsuccessful in many, and the time to drainage was prolonged. This may mean that the results appear consistent with those of the PTGBD group, despite the higher proportion of less severe cases. The PTBD group can have a higher CCI than the PTGBD group because of the higher number of patients with malignant diseases. There were no statistically significant differences. However, it should not be underestimated that the PTGBD group was more likely to have a higher technical success rate and a lower incidence rate of accidents.

The greatest advantage of PTGBD is its ease of use and high technical success rate. PTGBD and PTBD should be used interchangeably in different cases. However, in cases where PTBD is challenging to perform, PTGBD should be considered as an alternative treatment.

The present study had several limitations. First, it was a retrospective study conducted at two centers, and the number of cases was small. The two groups differed in terms of clinical characteristics, with the PTGBD group having milder jaundice. Moreover, PTBD was the only feasible option for cases who had undergone cholecystectomy. This may have introduced bias related to anatomical context. Second, evaluation of respiratory-related variability of positioning of the bile ducts and its influence on technique choice was at the discretion of the surgeon, potentially impacting the choice between PTGBD and PTBD. Future studies comparing PTGBD and PTBD under identical conditions should be performed.

## Conclusions

The outcomes of PTGBD are comparable with those of PTBD in patients with obstructive jaundice. Thus, PTGBD is a reasonable treatment option in cases of obstructive jaundice in which the intrahepatic bile duct dilatation is poor or PTBD is challenging to perform due to the patient’s general condition.

## Supporting information

S1 Table The data for each patient. PTGBD: percutaneous transhepatic gallbladder drainage. PTBD: percutaneous transhepatic biliary drainage. CCI: Charlson Comorbidity Index. T bil: Total bilirubin.
(PDF)
