# Supplementary material for: Outcomes of percutaneous transhepatic gallbladder drainage versus percutaneous transhepatic biliary drainage for obstructive jaundice
Source: PLoS One. 2025 Feb 24;20(2):e0310469. doi: 10.1371/journal.pone.0310469 (PMC11849854; doi:10.1371/journal.pone.0310469)
Supplement: S1 Table — PTGBD: percutaneous transhepatic gallbladder drainage. PTBD: percutaneous transhepatic biliary drainage. CCI: Charlson Comorbidity Index. T bil: Total bilirubin. (PDF) [file pone.0310469.s001.pdf]

patient number	PTGBD or PTBD	technical failure or success	CCI	use of antithrombotics medication	etiology of obstruction	severity	reson for PTBD or PTGBD	time to procedure (min)	early complication	late complication	baseline T.bil (mg/dl)	T.bil at day3 (mg/dl)	T.bil at day7(mg/dl)	T.bil at day14 (mg/dl)	duration of antibiotics therapy (days)	length of hospital stay (days)
1	PTGBD	success	5	no	benign	mile or moderate	failed cannulation	6	-	-	3	1.2	1.2	N/A	16	22
2	PTGBD	success	5	no	malignant	severe	failed cannulation	10	-	-	11.1	8.7	6.3	4.6	28	28
3	PTGBD	success	10	no	malignant	mile or moderate	surgical altered anatomy	4	-	-	11	8.78	6.66	5.67	18	65
4	PTGBD	success	7	yes	malignant	mile or moderate	failed cannulation	12	-	-	22.7	21.6	N/A	N/A	21	25
5	PTGBD	success	7	yes	benign	severe	respiratory failure or shock	12	-	-	5.8	1.6	1.3	0.8	14	23
6	PTGBD	success	6	no	benign	severe	respiratory failure or shock	4	-	-	4.9	2	3.1	6.1	27	40
7	PTGBD	success	6	no	benign	severe	respiratory failure or shock	5	-	-	2.3	0.9	1.7	0.9	9	19
8	PTGBD	success	9	no	benign	severe	respiratory failure or shock	6	-	-	2.9	1.3	1.3	0.8	25	58
9	PTGBD	success	7	no	benign	severe	respiratory failure or shock	37	-	infection	4.5	4.6	3.1	2	13	14
10	PTGBD	success	8	yes	benign	severe	failed cannulation	17	-	-	2.5	0.8	0.7	0.3	16	23
11	PTGBD	success	5	no	benign	severe	surgical altered anatomy	3	-	-	6.1	1.8	N/A	N/A	14	22
12	PTGBD	success	6	no	benign	severe	surgical altered anatomy	13	shock grade Ⅱ	-	4.6	1.5	1.5	1.6	21	27
13	PTGBD	success	4	no	benign	mile or moderate	surgical altered anatomy	10	-	-	2.6	0.6	0.6	0.4	8	21
14	PTGBD	success	6	no	benign	severe	surgical altered anatomy	3	-	-	2.5	0.8	0.6	N/A	8	8
15	PTGBD	success	10	no	benign	mile or moderate	surgical altered anatomy	3	-	-	2.7	0.6	0.5	N/A	5	16
16	PTGBD	success	8	no	benign	severe	surgical altered anatomy	7	-	-	4.3	1.4	2.2	1.1	14	35
17	PTGBD	success	8	yes	benign	mile or moderate	surgical altered anatomy	3	-	-	2.4	0.9	0.5	N/A	16	24
18	PTGBD	failure	3	no	benign	mile or moderate	surgical altered anatomy	30	-	-	4.1	N/A	N/A	N/A	16	31
19	PTGBD	success	7	no	benign	mile or moderate	surgical altered anatomy	40	-	-	3	1	0.7	N/A	12	41
20	PTGBD	success	3	no	benign	severe	surgical altered anatomy	20	-	-	5	3.3	3	0.9	22	23
21	PTGBD	success	3	no	benign	severe	respiratory failure or shock	144	miglation	-	4.9	1.8	N/A	N/A	13	21
22	PTGBD	success	4	no	malignant	mile or moderate	duodenal invasion	70	miglation	-	5	1.6	N/A	N/A	14	63
23	PTGBD	success	4	no	malignant	mile or moderate	duodenal invasion	6	-	miglation	10.1	13.7	N/A	N/A	8	19
24	PTGBD	success	11	no	malignant	severe	duodenal invasion	4	-	-	3.1	1.4	1	0.8	13	23
25	PTGBD	success	12	yes	malignant	mile or moderate	surgical altered anatomy	10	-	-	2.4	1.8	1.6	1.2	14	25
26	PTGBD	success	12	no	malignant	mile or moderate	surgical altered anatomy	5	-	-	4.2	1.4	N/A	N/A	16	21
27	PTGBD	success	9	no	malignant	mile or moderate	surgical altered anatomy	7	-	-	6.4	2.3	2	1.7	12	23
28	PTBD	success	7	no	benign	severe	surgical altered anatomy	30	-	obstruction	3.98	1.16	1.34	N/A	24	64
29	PTBD	success	7	yes	malignant	mile or moderate	surgical altered anatomy	14	-	-	9.23	6.15	5.38	4.56	2	57
30	PTBD	success	7	yes	malignant	mile or moderate	failed cannulation	56	bleeding	-	14.98	6.92	6.68	N/A	6	30
31	PTBD	success	4	no	malignant	mile or moderate	failed cannulation	293	-	-	21.9	18.3	17.7	15.4	13	24
32	PTBD	success	11	no	malignant	severe	duodenal invasion	74	-	obstruction	13.65	6.46	5.65	N/A	18	126
33	PTBD	success	9	no	malignant	severe	surgical altered anatomy	21	-	-	12.4	7.5	7.8	N/A	37	120
34	PTBD	success	9	no	malignant	mile or moderate	surgical altered anatomy	13	-	-	16.6	11.9	11.2	8.8	16	44
35	PTBD	success	9	no	malignant	mile or moderate	surgical altered anatomy	336	-	-	26.2	13	8.7	7.1	26	18
36	PTBD	success	9	no	malignant	mile or moderate	failed cannulation	242	-	-	11.5	8.6	7.9	5.9	20	40
37	PTBD	success	5	no	malignant	mile or moderate	surgical altered anatomy	28	-	-	11	6.8	6	5	9	18
38	PTBD	success	10	no	malignant	mile or moderate	failed cannulation	6	-	-	5.8	2	1.8	N/A	0	30
39	PTBD	success	5	no	malignant	mile or moderate	failed cannulation	54	-	-	25	18	17	13.4	5	81
40	PTBD	success	9	yes	malignant	mile or moderate	failed cannulation	51	-	-	2	1.1	0.8	0.9	5	54
41	PTBD	success	7	no	benign	mile or moderate	surgical altered anatomy	103	-	-	2.8	1.1	0.8	1.1	29	94
42	PTBD	success	8	no	benign	severe	failed cannulation	72	shock grade Ⅱ	-	4.9	1.2	2.2	2.2	11	79
43	PTBD	success	6	no	malignant	mile or moderate	failed cannulation	175	-	-	6.5	N/A	1.7	1.3	10	15
44	PTBD	success	6	no	malignant	mile or moderate	failed cannulation	169	-	-	11.9	11.8	7.6	4.8	4	129
45	PTBD	success	10	no	malignant	mile or moderate	duodenal invasion	173	miglation	-	6.3	4	2.5	3.1	26	84
46	PTBD	success	11	yes	malignant	mile or moderate	duodenal invasion	75	-	-	8.6	2.8	1.8	1.3	21	57
47	PTBD	success	11	no	malignant	mile or moderate	duodenal invasion	287	miglation	-	5.7	2.4	2.2	N/A	16	59
48	PTBD	success	9	no	malignant	mile or moderate	duodenal invasion	70	-	-	24.9	21.2	20.9	14.5	25	59
49	PTBD	success	10	no	malignant	severe	failed cannulation	144	miglation	-	4.7	3.3	1.9	N/A	8	26
50	PTBD	success	8	no	malignant	mile or moderate	surgical altered anatomy	150	-	-	8.5	3.9	3.5	3.6	8	64
51	PTBD	failure	8	no	malignant	mile or moderate	failed cannulation	75	-	-	8.1	N/A	N/A	N/A	7	25
52	PTBD	failure	10	no	benign	mile or moderate	failed cannulation	280	-	-	9.25	N/A	N/A	N/A	18	45
53	PTBD	failure	11	no	benign	mile or moderate	surgical altered anatomy	151	-	-	14.5	N/A	N/A	N/A	11	49
54	PTBD	failure	10	no	malignant	mile or moderate	surgical altered anatomy	60	-	-	9.4	N/A	N/A	N/A	13	54
55	PTBD	failure	8	no	malignant	mile or moderate	failed cannulation	80	-	-	13.5	N/A	N/A	N/A	11	57
